# Weight Gain After Smoking Cessation and Cancer Risk in 3 Prospective Cohorts in the United States

**DOI:** 10.1093/jncics/pkac005

**Published:** 2022-01-26

**Authors:** Yang Hu, Geng Zong, Qi Sun, Edward Giovannucci, Mingyang Song

**Affiliations:** 1 Department of Nutrition, Harvard T.H. Chan School of Public Health, Boston, MA, USA; 2 CAS Key Laboratory of Nutrition, Metabolism and Food Safety, Shanghai Institute of Nutrition and Health, University of Chinese Academy of Sciences, Chinese Academy of Sciences, Shanghai, China; 3 Channing Division of Network Medicine, Department of Medicine, Brigham and Women’s Hospital and Harvard Medical School, Boston, MA, USA; 4 Department of Epidemiology, Harvard T.H. Chan School of Public Health, Boston, MA, USA; 5 Clinical and Translational Epidemiology Unit, Massachusetts General Hospital and Harvard Medical School, Boston, MA, USA; 6 Division of Gastroenterology, Massachusetts General Hospital and Harvard Medical School, Boston, MA, USA

## Abstract

**Background:**

It remains unknown how cancer risks vary by duration of smoking cessation and whether the benefit is attenuated by postcessation weight gain.

**Methods:**

We prospectively followed 198* *565 persons from the Nurses’ Health Study (1978-2016), Nurses’ Health Study II (1991-2017), and Health Professionals Follow-up Study (1988-2016) who were free of cancer at baseline. We used proportional hazard Cox models to compare cancer risk between current smokers and former smokers with different durations of smoking cessation and postcessation weight gains.

**Results:**

During 4* *718* *199 person-years of follow-up, we identified 32* *456 cases of total cancer. Compared with current smokers, the risks for total and smoking-related cancer in past smokers were reduced to the level similar to never smokers after abstaining smoking for more than 26 years, with the hazard ratio of 0.69 (95% confidence interval [CI] = 0.63 to 0.76) for total cancer and 0.31 (95% CI = 0.26 to 0.37) for smoking-related cancer, whereas no risk reduction was found for obesity-related cancer. Comparing former smokers with current smokers, the multivariable-adjusted hazard ratios for postcessation weight gain of 0-4.9 kg, 5-9.9 kg, and 10 kg or higher were 0.85 (95% CI = 0.81 to 0.89), 0.88 (95% CI = 0.83 to 0.93), and 0.93 (95% CI = 0.88 to 1.00) for total cancer and 0.62 (95% CI = 0.58 to 0.67), 0.65 (95% CI = 0.60 to 0.71), and 0.71 (95% CI = 0.65 to 0.78) for total smoking-related cancer. In contrast, higher weight gain following smoking cessation was associated with a modest increased obesity-related cancer risk.

**Conclusion:**

Smoking cessation overall has a strong net association with lower risk of total cancer irrespective of weight gain. However, this inverse association may be attenuated by substantial postcessation weight gain, largely because of an increased risk of obesity-related cancers.

Smoking cessation has been shown to lower risks for many cancers relative to continuing smokers and to improve survival and quality of life among cancer patients (1); however, fewer than 10% of smokers succeed in quitting each year in the United States (2). One major hindrance to a smoker’s cessation attempt is the concern about weight gain following smoking cessation (3). It is estimated that former smokers gained 4.5-6.0 kg on average within 1 year after smoking cessation (3,4). Previous studies suggest a short-term increased risk of type 2 diabetes following smoking cessation because of substantial weight gain (5,6). However, how smoking cessation–related weight gain may influence cancer risk remains unknown.

Excess adiposity, as a possible promotor of tumorigenesis (7), is an established risk factor for at least 11 types of cancers, particularly endometrial cancer, postmenopausal breast cancer, pancreatic cancer, liver cancer, colorectal cancer, esophagus adenocarcinoma, and renal-cell kidney cancer (8). Some but not all of these obesity-related cancers are strongly associated with cigarette smoking, such as pancreatic and kidney cancer. Therefore, the effect of smoking cessation on subsequent cancer risk may vary by cancer types and depend, to some degree, on accompanied weight gain. How short-term weight gain may influence the long-term benefit of smoking cessation for risk of total cancer and individual cancers remains unknown. A comprehensive assessment of the net effect on cancer risk of smoking cessation after accounting for postcessation weight gain is warranted.

Therefore, we comprehensively examined the influence of postcessation weight gain on long-term cancer risks by leveraging data from the Nurses’ Health Study (NHS), NHSII, and Health Professionals Follow-up Study (HPFS)—3 large, well-characterized prospective cohort studies with repeated assessments of smoking status and body weight over 3 decades of follow-up. We assessed the risk trajectories for multiple composite cancer outcomes as well as major individual cancers among former smokers with different levels of weight gain.

## Methods

### Study Population

In 1976, the NHS was initiated by recruiting 121* *700 female registered nurses aged 30-55 years. With a similar study design, the HPFS was initiated in 1986 and included 51* *529 male health professionals aged 40-75 years, and the NHSII enrolled 116* *340 eligible female nurses aged 25-42 years in 1991. Participants from 3 cohorts were followed biennially through mailed questionnaires to inquire and update their demographic and lifestyle information as well as to identify incident diseases. The cumulative response rates in 3 cohorts exceeded 90% (9,10).

In this study, we set the baseline at the first follow-up cycle since recruitment for the HPFS (1988) and NHSII (1991) to identify incident former smokers. For the NHS, we used 1984 as the baseline when a comprehensive food frequency questionnaire was first used to collect dietary information. Participants who reported as past smokers at the cohorts’ recruitment were excluded from the analysis because their quitting status and weight change information were not available. We also excluded participants with prevalent cancer at baseline or those who did not return follow-up questionnaires. After exclusion, 84* *280 women in the NHS, 90* *037 women in the NHS II, and 24* *248 men in the HPFS were included in the analysis (Supplementary Figure 1, available online).

The study protocol was approved by the institutional review boards of the Brigham and Women’s Hospital and Harvard T.H. Chan School of Public Health and those of participating registries as required. The return of a completed questionnaire was considered informed consent.

### Assessment of Smoking Status and Weight Change

In the biennial follow-up questionnaires, participants were asked about their body weight; smoking status; and, for smokers, the number of cigarettes smoked during the last 2 years. In these cohorts, self-reported smoking status and body weight have been demonstrated to be highly accurate (11,12). We focused on weight change within the first 6 years of quitting smoking because our previous study showed that weight change in this time window was most pertinent to smoking cessation in the 3 cohorts (Supplementary Table 1, Supplementary Methods, available online) (5).

### Assessment of Cancer Incidence

Total cancer included all types of cancer with International Classification of Diseases 9th edition codes between 140 and 239 except nonmelanoma skin cancer. We also excluded nonfatal prostate cancer because of its high incidence in old men and the concerns for detection bias because of the prevalent use of prostate-specific antigen screening. The composite cancer outcomes included total smoking-related cancer, robust smoking-related cancer, and obesity-related cancer (Supplementary Methods, available online). Cancer diagnosis was confirmed by medical record review among participants who reported a diagnosis of cancer on the biennial follow-up questionnaires, and the study outcomes in the current study were all confirmed cancer cases.

### Assessment of Covariates

A validated semiquantitative food frequency questionnaire was administered to collect information of diet and alcohol intake every 4 years since 1984 in NHS, 1986 in HPFS, and 1991 in NHSII. Overall diet quality was assessed using the Alternative Health Eating Index score, which summarizes the consumption of 11 food or nutrients that are predictive of lower risk of multiple chronic diseases (13). Recreational physical activity was assessed using a validated questionnaire. We calculated weekly energy expenditure in metabolic equivalent hours to represent the total recreational physical activity level (14). Self-reported physical activity has been validated previously in our cohorts (15-19). Race information was self-reported by the participants in the follow-up questionnaire.

### Statistical Analysis

The primary exposures include smoking cessation and postcessation weight gain. The secondary outcomes are major individual cancers that have a relatively high incidence, including lung cancer, colorectal cancer, pancreatic cancer, kidney cancer, bladder cancer, postmenopausal breast cancer, ovarian cancer, and endometrial cancer. For each participant, person-time was counted from the return of baseline questionnaire to the date of cancer diagnosis, last return of a valid follow-up questionnaire, or the end of follow-up (June 2016 for the NHS, January 2016 for the HPFS, and June 2017 for the NHSII), whichever happened first.

We considered 2 multivariable-adjusted Cox models (Supplementary Methods, available online). Model 1 was adjusted for several time-invariant variables including cohort origin, race, and baseline body mass index and time-varying covariates, including history of hypertension, history of high cholesterol, family history of cancer, total energy, physical activity, multivitamin use, alcohol intake, Alternative Health Eating Index, and postmenopausal hormone use, whose information was updated every 2-4 years during the follow-up. To account for the history of smoking intensity, model 2 was further adjusted for cigarettes smoked per day and age at starting smoking. For this model, the effect estimates were missing for never smokers because they had no smoking history.

We used restricted cubic spline regression to assess cancer risk according to the duration of smoking cessation as a continuous variable (Supplementary Methods, available online) (20,21).We also categorized the duration of smoking cessation in a secondary analysis. To assess the joint association of smoking cessation and postcessation weight gain, we conducted a spline analysis for the duration of smoking cessation among former smokers with different levels of weight gain (0-4.9 kg, 5-9.9 kg, and ≥10 kg). Likelihood ratio tests were used to calculate the *P* values for interaction by comparing the model with spline terms of smoking cessation only and the model additionally adjusted for the product term between spline variables and continuous postcessation weight gain. Moreover, to assess whether former smokers with no weight change had different cancer risks from those with weight gain, we performed a sensitivity analysis to further categorize the 0-4.9 kg group into 2 subgroups: no weight change and weight gain of no more than 5 kg. Finally, we assessed cancer risk among former smokers with postcessation weight loss. All statistical tests were 2-sided at the statistical significance level of .05 and performed using SAS 9.4 (SAS Institute, Cary, NC).

## Results

Table 1 presents the age-standardized characteristics of study participants by sex according to smoking status and postcessation weight gain. Former smokers with higher postcessation weight gain were slightly younger and less physically active and had a higher prevalence of hypertension and hypercholesterolemia, lower alcohol consumption, and higher baseline body mass index. They also smoked more cigarettes per day and started smoking earlier than former smokers who gained less weight.

**Table:** **Table 1**. Age-standardized characteristics of study participants in 3 cohorts according to smoking status and weight gain following smoking cessation in the NHS (1978-2016), NHSII (1991-2017), and HPFS (1988-2016)^a^

Characteristics	Current smokers	Weight gain within 6 years after smoking cessation^b^			Never smokers
		0-4.9 kg	5-9.9 kg	≥10 kg	
Person-year	659* *668	299* *526	161* *033	123* *179	3* *470* *308
Age, mean (SD), y^b^	55.5 (11.2)	62.5 (11.9)	62.7 (11.6)	61.1 (11.3)	55.6 (12.8)
Baseline body mass index, mean (SD), kg/m²	23.9 (4.3)	23.2 (3.9)	23.9 (4.1)	26.1 (5.5)	24.6 (4.8)
Race, %					
African American	1.0	1.0	1.0	1.0	1.6
Asian	1.0	1.0	1.0	0.9	1.7
Others	1.1	1.0	1.0	1.0	1.5
White	97.0	97.1	97.1	97.0	95.2
Self-reported hypertension, %	27.2	30.7	35.6	43.6	31.2
Self-reported high cholesterol, %	33.7	39.6	45.6	49.9	39.3
Family history of cancer, %	45.3	50.1	51.6	51.5	51.1
Multivitamin use, %	31.8	41.6	42.7	41.5	43.1
Cigarettes smoked per day, %					
1-4	10.6	26.6	17.5	12.7	0.0
5-14	27.7	29.2	28.6	24.4	0.0
15-24	36.2	25.7	34.1	37.3	0.0
25-34	12.3	6.6	9.7	12.8	0.0
35-44	5.0	2.4	3.7	5.4	0.0
≥45	1.1	0.5	0.7	1.4	0.0
Unknown	7.2	9.0	5.7	6.0	100.0
Age at starting smoking, %, y					
<15	7.6	6.4	7.1	8.3	—
15-19	52.5	52.9	54.6	54.2	—
20-29	37.1	37.1	35.6	34.9	—
≥30	2.7	3.6	2.7	2.5	—
Physical activity, median (IQR) MET-h/wk	10.4 (3.0-32.4)	15.9 (5.3-37.7)	12.7 (4.0-32.2)	10.2 (2.9-29.3)	16.7 (5.6-43.6)
Alternative healthy eating index, mean (SD)	47.8 (9.7)	52.1 (9.9)	50.9 (9.7)	50 (9.6)	51.3 (10.3)
Alcohol consumption median (IQR), g/day	2.9 (0.5-11.2)	3.7 (0.9-10.4)	3.0 (0.7-9.1)	1.9 (0.3-6.6)	1.1 (0-4.7)
Total energy intake, mean (SD), Kcal/d	1747 (576)	1743 (553)	1748 (553)	1761 (576)	1802 (575)

a Data were based on total cancer analysis. HPFS = Health Professionals Follow-up Study; IQR = interquartile range; MET = metabolic equivalent; NHS = Nurses’ Health Study; NHSII = Nurses’ Health Study II.

b Value is not age adjusted.

**Figure 1.**
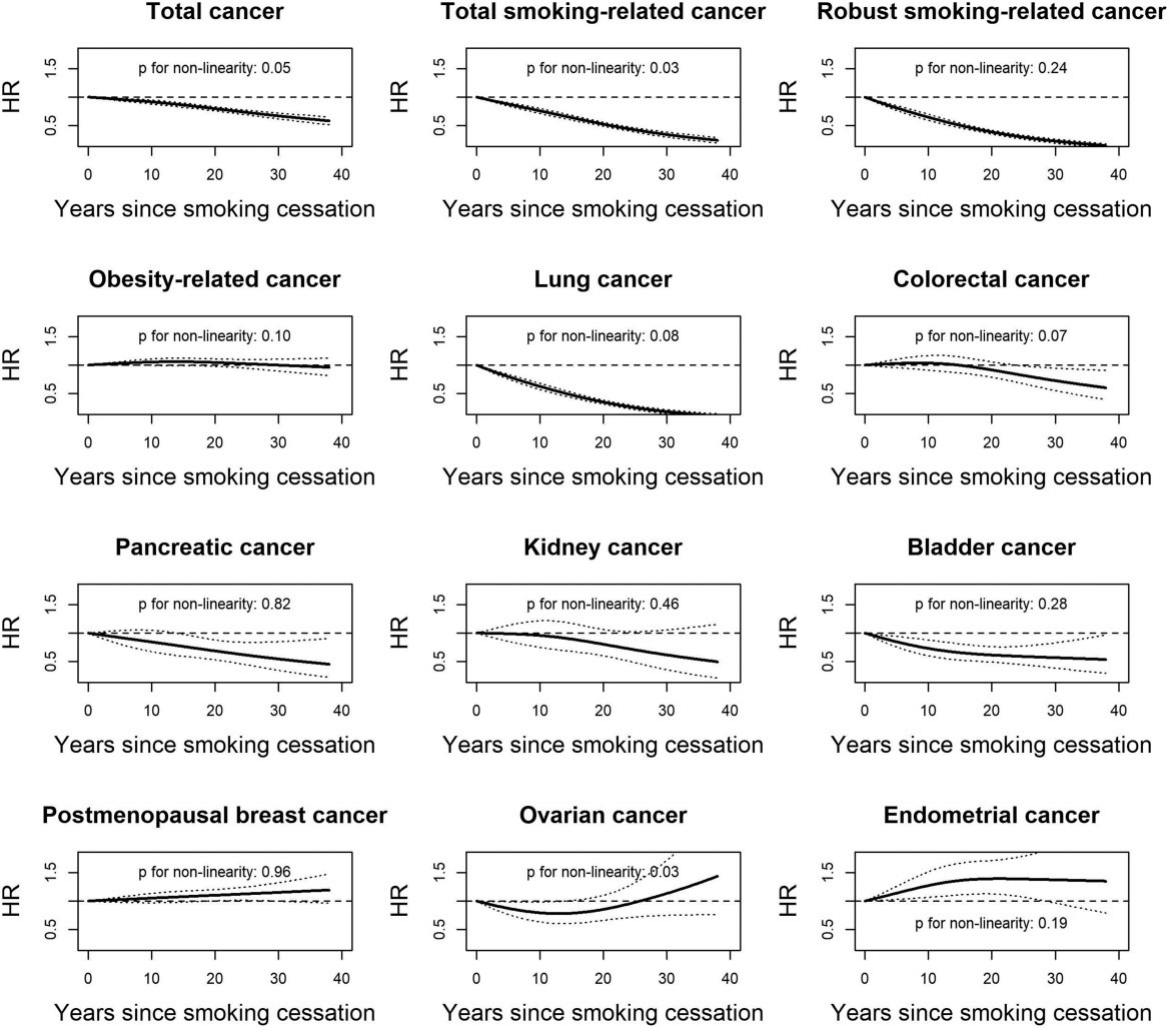
Association between duration of smoking cessation and cancer risks. Models were age- (months) and calendar-time stratified and adjusted for cohort origin (Nurses’ Health Study, Nurses’ Health Study II, Professionals Follow-up Study), race (African American, Asian, Others, White), history of hypertension, history of high cholesterol, family history of cancer, total energy (quintiles), physical activity (quintiles), multivitamin use (yes, no), alcohol intake (none, 1-4, 5-9, 10-14, 15-29 g, ≥30 grams per day), Alternative Health Eating Index (quintiles), baseline body mass index (<21.0, 21.0-22.9, 23.0-24.9, 25.0-26.9, 27.0-29.9, 30.0-32.9, 33.0-34.9, or ≥35.0 kg/m^2^), cigarettes smoked per day (1-4, 5-14, 15-24, 25-34, 35-44, ≥45, unknown), and age at starting smoking (younger than 15, 15-19, 20-29, 30-39, 40-49, 50-59, 60 years and older). The 2-sided likelihood ratio test was used to calculate the *P* values for nonlinearity. Total smoking-related cancer includes liver cancer, colorectal cancer, lung cancer, oral cavity and throat cancer, esophagus cancer, larynx cancer, stomach cancer, pancreatic cancer, bladder cancer, kidney cancer, cervix cancer, and acute myeloid leukemia. Robust smoking-related cancer includes oral cavity and throat cancer, lung cancer, esophagus cancer, and bladder cancer. Obesity-related cancer includes esophagus cancer, liver cancer, kidney cancer, myeloma, pancreatic cancer, colorectal cancer, gallbladder cancer, postmenopausal breast cancer, ovarian cancer, endometrial cancer, fatal prostate cancer, and thyroid cancer. HR = hazard ratio.

During 4* *713* *714 person-years of follow-up, we documented 32* *456 cancer cases, among whom there were 9666 (29.8%) smoking-related cancers and 18* *460 (56.9%) obesity-related cancers. Compared with current smokers, former smokers with longer cessation duration had a monotonically decreased risk for total cancer, total smoking-related cancer, and robust smoking-related cancer but similar stable risks for obesity-related cancer (Figure 1). The risks were reduced to the similar level as never smokers after 26 years of smoking cessation for total and smoking-related cancer. In comparison with current smokers, the multivariable-adjusted hazard ratio (HR) for total cancer decreased from 1.01 (95% confidence interval [CI] = 0.93 to 1.09) in 2 years of quitting to 0.69 (95% CI = 0.63 to 0.76) of more than 26 years of cessation; for total smoking-related cancer, the corresponding hazard ratio decreased from 0.83 (95% CI = 0.73 to 0.93) to 0.31 (95% CI = 0.26 to 0.37); for robust smoking-related cancer, the hazard ratio decreased from 0.69 (95% CI = 0.58 to 0.81) to 0.20 (95% CI = 0.16 to 0.25); and for obesity-related cancer, it changed from 1.27 (95% CI = 1.14 to 1.41) to 1.12 (95% CI = 0.98 to 1.27) (Supplementary Table 2, available online). The risk-decreasing trajectories were observed for most individual cancers except postmenopausal breast and endometrial cancers, for which the risks were statistically significantly increased compared with current smokers. The risk for ovarian cancers decreased in the first 15 years of smoking cessation duration, after which the risk appeared to gradually increase during longer follow-up (*P* for nonlinearity = 0.03).

During 4* *718* *199 person-years of follow-up, we identified 32* *456 cases of total cancer. Substantial weight gain following smoking cessation slightly attenuated the inverse association for total cancer but not smoking-related cancer (Table 2). Compared with current smokers, the hazard ratios of total cancer were 0.85 (95% CI = 0.81 to 0.89) for former smokers who gained 0-4.9 kg body weight within 6 years of smoking cessation, 0.88 (95% CI = 0.83 to 0.93) for former smokers who gained 5-9.9 kg, and 0.93 (95% CI = 0.88 to 1.00) for those who gained 10 kg or more. The corresponding estimates for total smoking-related cancer were 0.62 (95% CI = 0.58 to 0.67), 0.65 (95% CI = 0.60 to 0.71), and 0.71 (95% CI = 0.65 to 0.78), respectively. For robust smoking-related cancer, the risk estimates were 0.50 (95% CI = 0.46 to 0.55), 0.53 (95% CI = 0.47 to 0.59), and 0.53 (95% CI = 0.47 to 0.61), respectively.

**Table 2.** Pooled hazard ratios (95% CIs) of associations between weight gain following smoking cessation and risk of cancers

Cancer outcomes	Current smokers	Weight gain within 6 years after smoking cessation^a^			Never smokers
		0-4.9 kg	5-9.9 kg	≥10 kg	
Total cancer					
Case/person-year	5927/659* *668	3076/299* *526	1792/161* *033	1366/123* *179	20* *295/3* *470* *308
Age-adjusted model	1.00	0.88 (0.84 to 0.92)	0.94 (0.89 to 0.99)	1.00 (0.94 to 1.06)	0.69 (0.67 to 0.71)
Multivariable-adjusted model 1^b^	1.00	0.85 (0.82 to 0.89)	0.90 (0.85 to 0.95)	0.95 (0.89 to 1.01)	0.68 (0.66 to 0.71)
Multivariable-adjusted model 2^c^	1.00	0.85 (0.81 to 0.89)	0.88 (0.83 to 0.93)	0.93 (0.88 to 1.00)	
Total smoking-related cancer^d^					
Case/person-year	2965/662* *286	1183/301* *188	720/161* *985	551/123* *885	4247/3* *485* *136
Age-adjusted model	1.00	0.59 (0.55 to 0.63)	0.67 (0.62 to 0.73)	0.76 (0.70 to 0.84)	0.28 (0.27 to 0.30)
Multivariable-adjusted model 1^b^	1.00	0.60 (0.56 to 0.65)	0.68 (0.62 to 0.73)	0.75 (0.69 to 0.83)	0.29 (0.28 to 0.31)
Multivariable-adjusted model 2^c^	1.00	0.62 (0.58 to 0.67)	0.65 (0.60 to 0.71)	0.71 (0.65 to 0.78)	
Robust smoking-related cancer^e^					
Case/person-year	2039/662* *964	675/301* *566	429/162* *216	304/124* *075	1092/3* *487* *652
Age-adjusted model	1.00	0.45 (0.41 to 0.50)	0.54 (0.48 to 0.60)	0.56 (0.50 to 0.64)	0.10 (0.09 to 0.11)
Multivariable-adjusted model 1^b^	1.00	0.46 (0.42 to 0.51)	0.55 (0.49 to 0.61)	0.59 (0.52 to 0.67)	0.11 (0.10 to 0.12)
Multivariable-adjusted model 2^c^	1.00	0.50 (0.46 to 0.55)	0.53 (0.47 to 0.59)	0.53 (0.47 to 0.61)	
Obesity-related cancer^f^					
Case/person-year	2714/661* *933	1753/300* *522	1046/161* *568	811/123* *588	12* *136/3* *477* *419
Age-adjusted model	1.00	1.05 (0.99 to 1.12)	1.14 (1.07 to 1.23)	1.25 (1.15 to 1.35)	0.97 (0.93 to 1.01)
Multivariable-adjusted model 1^b^	1.00	1.02 (0.96 to 1.09)	1.09 (1.01 to 1.17)	1.15 (1.06 to 1.24)	0.94 (0.90 to 0.98)
Multivariable-adjusted model 2^c^	1.00	1.05 (0.98 to 1.12)	1.11 (1.03 to 1.20)	1.20 (1.10 to 1.30)	
Lung cancer					
Case/person-year	1640/663* *307	525/301* *703	341/162* *293	244/124* *130	489/3* *488* *189
Age-adjusted model	1.00	0.42 (0.38 to 0.47)	0.51 (0.45 to 0.58)	0.54 (0.47 to 0.62)	0.06 (0.05 to 0.06)
Multivariable-adjusted model 1^b^	1.00	0.44 (0.40 to 0.49)	0.53 (0.47 to 0.60)	0.57 (0.50 to 0.66)	0.06 (0.06 to 0.07)
Multivariable-adjusted model 2^c^	1.00	0.48 (0.43 to 0.53)	0.51 (0.45 to 0.58)	0.52 (0.45 to 0.59)	
Colorectal cancer					
Case/person-year	483/663* *883	292/301* *824	156/162* *383	150/124* *148	1713/3* *487* *019
Age-adjusted model	1.00	0.99 (0.86 to 1.15)	0.98 (0.82 to 1.18)	1.45 (1.20 to 1.75)	0.76 (0.68 to 0.84)
Multivariable-adjusted model 1^b^	1.00	1.01 (0.87 to 1.17)	0.98 (0.81 to 1.18)	1.39 (1.15 to 1.68)	0.77 (0.69 to 0.86)
Multivariable-adjusted model 2^c^	1.00	0.99 (0.85 to 1.16)	0.94 (0.78 to 1.14)	1.35 (1.11 to 1.64)	
Pancreatic cancer					
Case/person-year	161/664* *219	85/302* *034	50/162* *492	41/124* *268	480/3* *488* *325
Age-adjusted model	1.00	0.68 (0.52 to 0.88)	0.77 (0.55 to 1.06)	0.90 (0.64 to 1.28)	0.52 (0.43 to 0.63)
Multivariable-adjusted model 1^b^	1.00	0.69 (0.53 to 0.90)	0.76 (0.55 to 1.06)	0.86 (0.60 to 1.22)	0.52 (0.43 to 0.63)
Multivariable-adjusted model 2^c^	1.00	0.77 (0.58 to 1.02)	0.85 (0.61 to 1.19)	0.95 (0.66 to 1.36)	
Kidney cancer					
Case/person-year	134/664* *169	69/302* *013	49/162* *476	30/124* *256	492/3* *488* *077
Age-adjusted model	1.00	0.81 (0.60 to 1.09)	1.05 (0.75 to 1.46)	0.94 (0.63 to 1.41)	0.74 (0.60 to 0.90)
Multivariable-adjusted model 1^b^	1.00	0.83 (0.62 to 1.12)	1.00 (0.72 to 1.40)	0.77 (0.51 to 1.15)	0.69 (0.56 to 0.85)
Multivariable-adjusted model 2^c^	1.00	0.90 (0.65 to 1.23)	1.09 (0.77 to 1.54)	0.85 (0.56 to 1.30)	
Bladder cancer					
Case/person-year	262/664* *038	113/301* *957	67/162* *458	44/124* *245	394/3* *488* *189
Age-adjusted model	1.00	0.64 (0.51 to 0.80)	0.72 (0.55 to 0.95)	0.71 (0.52 to 0.99)	0.27 (0.23 to 0.32)
Multivariable-adjusted model 1^b^	1.00	0.60 (0.48 to 0.75)	0.68 (0.52 to 0.90)	0.69 (0.50 to 0.96)	0.27 (0.22 to 0.32)
Multivariable-adjusted model 2^c^	1.00	0.65 (0.51 to 0.83)	0.69 (0.52 to 0.92)	0.68 (0.48 to 0.96)	
Postmenopausal breast cancer					
Case/person-year	1277/618* *458	927/281* *178	578/154* *523	405/119* *525	5820/3* *095* *383
Age-adjusted model	1.00	1.14 (1.05 to 1.24)	1.27 (1.15 to 1.41)	1.23 (1.09 to 1.37)	1.05 (0.99 to 1.12)
Multivariable-adjusted model 1^b^	1.00	1.06 (0.98 to 1.16)	1.17 (1.06 to 1.30)	1.15 (1.02 to 1.29)	1.04 (0.97 to 1.10)
Multivariable-adjusted model 2^c^	1.00	1.09 (1.00 to 1.20)	1.19 (1.07 to 1.32)	1.19 (1.06 to 1.34)	
Ovarian cancer					
Case/person-year	217/619* *472	108/281* *963	58/155* *039	52/119* *842	857/3* *100* *249
Age-adjusted model	1.00	0.88 (0.69 to 1.11)	0.84 (0.62 to 1.12)	1.10 (0.81 to 1.50)	0.90 (0.77 to 1.05)
Multivariable-adjusted model 1^b^	1.00	0.85 (0.67 to 1.08)	0.80 (0.60 to 1.08)	1.03 (0.76 to 1.40)	0.87 (0.74 to 1.01)
Multivariable-adjusted model 2^c^	1.00	0.84 (0.65 to 1.08)	0.80 (0.59 to 1.09)	1.10 (0.79 to 1.51)	
Endometrial cancer					
Case/person-year	262/619* *410	163/281* *893	113/154* *989	109/119* *788	1742/3099* *
Age-adjusted model	1.00	1.21 (0.99 to 1.47)	1.53 (1.22 to 1.91)	1.99 (1.59 to 2.50)	1.61 (1.41 to 1.84)
Multivariable-adjusted model 1^b^	1.00	1.22 (1.00 to 1.49)	1.44 (1.15 to 1.81)	1.56 (1.24 to 1.95)	1.43 (1.25 to 1.64)
Multivariable-adjusted model 2^c^	1.00	1.21 (0.98 to 1.50)	1.44 (1.14 to 1.82)	1.57 (1.23 to 2.00)	

a Weight changes stopped updating after 6 years of quitting. CI = confidence interval.

b Models were age- (months) and calendar-year stratified and adjusted for cohort origin (Nurses’ Health Study, Nurses’ Health Study II, Health Professionals Follow-up Study), race (White, African American, Asian, Others), history of hypertension, history of high cholesterol, family history of cancer, total energy (quintiles), physical activity (quintiles), multivitamin use (yes, no), alcohol intake (none, 1-4 , 5-9 , 10-14 , 15-29 , ≥30 g/d), Alternative Health Eating Index (quintiles), and baseline body mass index (<21.0, 21.0-22.9, 23.0-24.9, 25.0-26.9, 27.0-29.9, 30.0-32.9, 33.0-34.9, or ≥35.0 kg/m^2^). Postmenopausal hormone use was adjusted for women.

c Model 1 plus cigarettes smoked per day (1-4, 5-14, 15-24, 25-34, 35-44, ≥ 45, unknown), age at starting smoking (younger than 15, 15-19, 20-29, 30-39, 40-49, 50-59, 60 years or older). Never smokers were excluded in the model.

d Total smoking-related cancer includes liver cancer, colorectal cancer, lung cancer, oral cavity and throat cancer, esophagus cancer, larynx cancer, stomach cancer, pancreatic cancer, bladder cancer, kidney cancer, cervix cancer, and acute myeloid leukemia.

e Robust smoking-related cancer includes oral cavity and throat cancer, lung cancer, esophagus cancer, and bladder cancer.

f Obesity-related cancer includes esophagus cancer, liver cancer, kidney cancer, myeloma, pancreatic cancer, colorectal cancer, gallbladder cancer, postmenopausal breast cancer, ovarian cancer, endometrial cancer, fatal prostate cancer, and thyroid cancer.

For obesity-related cancer, we found an increased risk proportional to the level of postcessation weight gain: the hazard ratios were 1.05 (95% CI = 0.98 to 1.12), 1.11 (95% CI = 1.03 to 1.20), and 1.20 (95% CI = 1.10 to 1.30) for former smokers who gained 0-4.9 kg, 5-9.9 kg, and 10 kg or more, respectively. For individual cancers, higher weight gain was primarily associated with increased risk for colorectal cancer, breast cancer, and endometrial cancer. In the spline analysis stratified by postcessation weight gain, the risk of total cancer decreased faster among former smokers with less weight gain, whereas similar risk decreasing trend was found for total smoking-related cancer and robust smoking-related cancer among former smokers with different levels of weight gain (Supplementary Figure 2, A-C, available online). For obesity-related cancer, compared with current smokers, former smokers who gained 10 kg or more had a statistically significant higher risk, but those who gained less than 10 kg had no risk elevation (Supplementary Figure 2, D, available online). We did not detect any statistically significant interaction between smoking cessation duration and postcessation weight gain in the spline analysis, suggesting that the spline curves stratified by weight gain did not statistically significantly differ from each other in all 4 composite cancer outcomes.

In the sensitivity analysis, we found similar results between former smokers with no weight change and those with weight gain of up to 5 kg (Supplementary Table 3, available online). Former smokers who lost weight within 6 years of smoking cessation had a higher risk than current smokers for obesity-related cancer such as colorectal cancer, kidney cancer, breast cancer, and endometrial cancer (Supplementary Table 4, available online).

## Discussion

Leveraging data from 3 large prospective cohorts, we found that longer smoking cessation was associated with a lower risk of total cancer and smoking-related cancer in a dose-response manner compared with current smokers, and the risk for obesity-related cancer was not reduced after smoking cessation. Gaining 10 kg or more within 6 years of smoking cessation slightly attenuated the inverse association for total cancer but not smoking-related cancer. On the contrary, higher postcessation weight gain was associated with increased risk of obesity-related cancer, particularly colorectal cancer, breast cancer, and endometrial cancer. These results suggest that weight management is critical to enhance the benefit of smoking cessation and avoid potential risk elevation for obesity-related cancer.

Smoking cessation is an effective way to reduce cancer incidence. However, no study has yet comprehensively characterized the cancer risk trajectory according to duration of smoking cessation. Our data showed that the risk of total cancer was monotonically decreased after smoking cessation, and the risk for total smoking-related cancer decreased more rapidly. An even faster risk reduction was observed for robust smoking-related cancer, which encompassed cancers that were strongly associated with cigarette smoking. Notably, the risks for both total cancer and smoking-related cancer were reduced to the similar level as never smokers after abstaining smoking for more than 26 years. Lung cancer had the steepest slope of risk reduction, whereas the decreasing trends were similar for other individual cancers including pancreatic cancer, kidney cancer, and bladder cancer. On the other hand, we found the risk for obesity-related cancer was not reduced after smoking cessation and even increased for colorectal cancer, breast cancer, and endometrial cancer during the course of smoking cessation. This may be explained by the relative modest associations (HRs approximately 1.10-1.30) between smoking and risk of colorectal cancer and breast cancer (22,23), for which weight gain may play a more important role in determining the risk after smoking cessation. The increased risk found for endometrial cancer after smoking cessation was expected as previous epidemiological studies have established a robust inverse association between cigarette smoking and endometrial cancer risk, which may be related to the antiestrogenic effects of smoking (24).Weight gain following smoking cessation is usually the result of a positive energy balance due to increased appetite and reduced energy expenditure. Withdrawal from nicotine not only increases the rewarding value of food but also elevates the threshold of the reward in the brain, which collectively lead to high energy-dense food consumption (25). A number of interventions such as a low-calorie diet, drug treatment, exercise, and nicotine replacement therapy have proved to be effective in lowering postcessation weight gain in the short term; however, it remains uncertain if there is an optimal intervention strategy to prevent long-term weight gain without undermining the chance of achieving abstinence (26). In the stratified analysis by postcessation weight gain among those who had ceased smoking, the risk reduction for total cancer was attenuated from 10% for participants with less than 10 kg of weight gain to 4% for those with 10 kg or more weight gain. No statistically significant attenuations were found for total smoking-related cancer with a consistent approximation of 35% risk reduction across the categories of weight gain. These findings are generally consistent with a South Korean study (27), but we observed a much lower estimate of risk reduction, possibly because of the repeated assessment of smoking status and longer follow-up. In contrast to that study, we identified a dose-response pattern between higher postcessation weight gain and increased risk of obesity-related cancer among former smokers, although such positive associations varied among individual cancers, which may be explained by the different effects of weight gain on different cancers. In addition, the positive associations observed among participants with weight loss after smoking cessation may be a manifestation of reverse causation that patients having subclinical malignancies had already lost weight while quitting smoking.

The ensuing increased risk of obesity-related cancer after smoking cessation should not discourage smokers to quit smoking, because the benefit outweighs the risk for total cancer. Despite no clear risk reduction for breast, prostate, and colorectal cancers, previous studies have shown that smoking cessation may lower the mortality for these cancers (28–30). Moreover, compelling mechanistic data have demonstrated that various carcinogens in cigarettes such as nicotine can compromise innate immune system (31), promote tumor progression and metastasis (32), and increase angiogenesis and transformation (33), thereby resulting in more advanced stage at cancer diagnosis and higher mortality. Therefore, abstinence from smoking and quitting early should remain the key message for cancer prevention.

Experimental studies using murine models of human malignancies have shown that obesity is an important tumor promoter and may result in earlier appearance, greater frequency, accelerated growth, larger tumor size, and in some cases more frequent metastasis of genetically initiated tumors (7). It is biologically plausible that the rapidly accumulated fat following smoking cessation may induce a series of procancer metabolic and endocrine abnormalities, including alterations in sex hormone metabolism, insulin and insulin-like growth factor signaling, and chronic inflammation (34,35). Additional studies are warranted to elucidate the mechanisms associated with the net health effect of smoking cessation for individual cancers.

Large sample size, repeated measurement of smoking status and weight change, and long follow-up period are the main strengths of the current study. A major limitation of our analysis was that, without information on reasons for weight loss, we were unable to provide a robust estimate for postcessation weight loss because of the influence of reverse causality. Nevertheless, results from our sensitivity analysis showed that the potential reverse causality may have mainly affected certain obesity-related cancers but less so for total cancers and smoking-related cancers. Another limitation is that we were unable to study less common cancers because of small case numbers. In addition, there may be misclassification in our assessment for the timing of smoking cessation because we did not inquire the exact date of smoking cessation in the questionnaires but instead assessed smoking cessation based on whether participants changed from current to past smokers in consecutive questionnaires. However, such measurement errors are likely to be nondifferential with respect to the outcomes and thus have likely biased the associations toward the null. Last, our findings may largely pertain to the White female health professionals, and the generalizability to other populations with different characteristics may be limited. Nevertheless, the underlying biology linking smoking cessation, weight gain, and cancer development is unlikely to vary across different ethnic groups, sex, and professions.

In summary, we found that substantial weight gain slightly attenuated the strong beneficial association of smoking cessation with total cancer but not with smoking-related cancer and might increase risk of obesity-related cancers. Our data emphasize the importance of weight management following smoking cessation for improved cancer prevention.

## Funding

The cohorts were supported by grants UM1 CA186107, P01 CA87969, U01 CA167552, and U01 CA176726 from the National Institutes of Health; and MRSG-17-220-01—NEC from the American Cancer Society.

## Notes

**Role of the funders:** The National Institutes of Health had no role in the design, conduct, analysis, or reporting of this study. The funding sources did not participate in the design and conduct of the study; collection, management, analysis, and interpretation of the data; preparation, review, or approval of the manuscript; and decision to submit the manuscript for publication.

**Disclosures:** The authors have completed and submitted the ICMJE Form for Disclosure of Potential Conflicts of Interest, and none were reported.

**Author contributions:** YH, GZ, MYS: Conceptualization, Methodology; QS, EG, MYS: Data curation, Funding acquisition, Supervision, Writing-review & editing; YH: Formal analysis, Writing-original draft.

**Acknowledgements:** We thank the participants and staff of the NHS and HPFS for their valuable contributions as well as the following state cancer registries for their help: AL, AZ, AR, CA, CO, CT, DE, FL, GA, ID, IL, IN, IA, KY, LA, ME, MD, MA, MI, NE, NH, NJ, NY, NC, ND, OH, OK, OR, PA, RI, SC, TN, TX, VA, WA, WY. The authors assume full responsibility for analyses and interpretation of these data.

## Supplementary Material

pkac005_Supplementary_Data

## Data Availability

The data underlying this article will be shared on reasonable request to the corresponding author.
