# Supplementary material for: Weight Gain After Smoking Cessation and Cancer Risk in 3 Prospective Cohorts in the United States
Source: JNCI Cancer Spectr. 2022 Jan 26;6(1):pkac005. doi: 10.1093/jncics/pkac005 (PMC8882388; doi:10.1093/jncics/pkac005)
Supplement: pkac005_Supplementary_Data [file pkac005_supplementary_data.pdf]

Supplementary Materials

Supplementary Methods

Details of smoking status and weight change assessment

Incident former smokers were defined as participants who had reported that they were smokers in the previous cycle but as past smokers in the current cycle, assuming the beginning of the previous cycle as the onset of quitting. The duration of smoking cessation was calculated from the onset of quitting to the relapse of smoking or the end of follow-up, whichever occurred first. For individuals with missing smoking status in a particular follow-up cycle, we performed imputation based on their reports in the previous cycle if they had reported the same smoking status in at least two consecutive previous cycles. After imputation (3.5% of the total person-time), we excluded the person-time for participants with missing smoking status. We stopped updating the weight change information for participants who had ceased smoking for more than 6 years because of our focus on short-term post-cessation weight gain (**Supplementary Table 1**). We categorized former smokers according to their weight gain into three groups: 0-5 kg, >5-10 kg, and ≥ 10 kg. Missing values of body weight were carried forward with the last available value (5.4% of the total person-time). We excluded participants with weight loss in the analysis because individuals with subclinical cancer tend to lose weight and stop smoking, leading to reverse causation that could have biased the effect estimates for the weight loss group.[1] In addition, we conducted a sensitivity analysis among former smokers who lost weight within the first 6 years after smoking cessation.

Definitions of composite cancer outcomes

Total smoking-related cancer was defined as the malignant neoplasms that have been

causally related to cigarette smoking, including cancers in the liver, colon and rectum, lung, oral cavity and throat, esophagus, larynx, stomach, pancreas, bladder, kidney, and cervix, and acute myeloid leukemia.[2] Using the population-attributable risk fraction estimates by 2014 US Surgeon General's Report, we additionally defined robust smoking-related cancer (oral cavity and throat cancer, lung cancer, esophagus cancer, and bladder cancer) as those for which tobacco accounts for at least 40% of cases in U.S..[3] Obesity-related cancer (esophagus cancer, liver cancer, kidney cancer, myeloma, pancreatic cancer, colorectal cancer, gallbladder cancer, postmenopausal breast cancer, ovarian cancer, endometrial cancer, fatal prostate cancer, and thyroid cancer) was defined according to previous studies.[4]

Brief rationale and interpretation of Cox proportional hazard model

Because smoking status varied during the follow-up, we employed a time-dependent Cox proportional hazard model to account for this time-varying exposure. The models were stratified by both age (months) and calendar time to minimize their confounding effects. The hazard ratios (HRs) assessed the relative risks of developing cancers comparing former smokers having different weight gain with current smokers during the follow-up. The proportional hazards assumption was tested by including the interaction terms between exposure variables and follow-up time for all models and no significant violations were detected.

Brief rationale and interpretation of restricted cubic spline regression

To allow for a more flexible characterization of the cancer risk trajectory following smoking cessation, we used restricted cubic spline regression to model the duration of smoking cessation as a non-linear function. The parameters of the spline variables from the restricted cubic spline regression were entered into the Cox models in the main analysis to produce a smoothing curve that delineated the relationship between

smoking cessation duration and cancer risks. P values for non-linearity were calculated from the models to determine whether the overall associations were linear.

References

1. Kenfield SA, Stampfer MJ, Rosner BA, Colditz GA. Smoking and smoking cessation in relation to mortality in women. JAMA. 2008;299(17):2037–47.
2. United States Department of Health and Human Services, Centers for Disease Control and Prevention, National Center for Chronic Disease Prevention and Health Promotion, Office on Smoking and Health. The Health Consequences of Smoking—50 Years of Progress: A Report of the Surgeon General. 2014.
3. Giovannucci E. An integrative approach for deciphering the causal associations of physical activity and cancer risk: The role of adiposity. J Natl Cancer Inst. 2018;110(9):935–41.
4. Lauby-Secretan B, Scoccianti C, Loomis D, Grosse Y, Bianchini F, Straif K. Body Fatness and Cancer–Viewpoint of the IARC Working Group. N Engl J Med. 2016;375(8):794–8.

Supplementary Table 1. Illustration of definition of smoking cessation status and post-cessation weight change during follow-up.[a,b]

Smoking status	Follow-up cycles											Smoking cessation starting cycles	Smoking cessation duration, years	Weight change period
	1	2	3	4	5	6	7	8	9	10	11			
Never smokers	N	N	N	N	N	N	N	N	N	N	N	-	-	-
Former smokers who never relapsed	C	C	C	P	P	P	P	P	P	P	P	3	16	4-3, 5-3, 6-3 & 6-3 thereafter
Former smokers who relapsed	C	P	P	C	C	P	P	P	P	C	C	1 & 5	4 & 8	2-1, 3-1 for cycle 2 &3<br>6-5,7-5, 8-5, & 8-5 thereafter until cycle 9
Never smokers to smokers	N	N	N	N	C	C	C	C	C	C	C	-	-	-
Never smokers to smokers, and then to former smokers who never relapsed	N	N	N	N	N	N	C	C	P	P	P	8	6	9-8, 10-8, 11-8
Never smokers to smokers, and then to former smokers who relapsed	N	N	C	P	P	C	C	P	P	P	P	3 & 7	4 & 8	5-4, 6-4 for cycle 4 & 5<br>8-7, 9-7, 10-7, & 10-7 thereafter
Current smokers	C	C	C	C	C	C	C	C	C	C	C	-	-	-

[a]The letter C indicates current smokers, P for past smokers, and N for never smokers.
[b]For the simplicity of illustration, all types of participants were assumed to be followed until the end of study.

Supplementary Table 2. Pooled hazard ratios (95% CIs) for associations between smoking cessation duration and risk of cancers.[a]

Cancer outcomes	Current smokers	0 to 2 years	>2 to 4 years	>4 to 6 years	>6 to 8 years	>8 to 10 years	>10 to 18 years	>18 to 26 years	>26 years	Never smokers
Total cancer										
Case/person-year	5,927/659,668	718/82,894	780/87,517	755/79,898	738/76,547	726/69,379	2,258/214,944	1,456/123,296	576/49,619	20,295/3,470,308
Model 1[a]	1	0.96 (0.88,1.03)	0.93 (0.87,1.01)	0.95 (0.88,1.02)	0.93 (0.86,1.00)	0.94 (0.87,1.02)	0.84 (0.80,0.88)	0.82 (0.78,0.87)	0.78 (0.71,0.85)	0.68 (0.66,0.70)
Model 2[b]	1	1.01 (0.93,1.09)	0.97 (0.90,1.05)	0.99 (0.91,1.07)	0.95 (0.88,1.03)	0.95 (0.88,1.03)	0.82 (0.78,0.87)	0.76 (0.71,0.81)	0.69 (0.63,0.76)	–
Total smoking-related cancer[c]										
Case/person-year	2,965/662,286	293/83,280	349/87,876	370/80,225	316/76,908	303/69,783	902/216,151	560/124,076	156/49,956	4,247/3,485,136
Model 1[a]	1	0.70 (0.62,0.79)	0.76 (0.68,0.85)	0.83 (0.75,0.93)	0.71 (0.63,0.80)	0.70 (0.62,0.79)	0.58 (0.53,0.62)	0.53 (0.48,0.58)	0.36 (0.30,0.42)	0.29 (0.28,0.31)
Model 2[b]	1	0.83 (0.73,0.93)	0.86 (0.77,0.97)	0.94 (0.84,1.05)	0.78 (0.69,0.88)	0.74 (0.66,0.84)	0.58 (0.54,0.63)	0.49 (0.44,0.54)	0.31 (0.26,0.37)	-
Robust smoking-related cancer[d]										
Case/person-year	2,039/662,964	156/83,382	210/87,986	217/80,354	184/77,003	177/69,882	509/216,436	305/124,258	80/50,007	1,092/3,487,652
Model 1[a]	1	0.55 (0.47,0.65)	0.67 (0.58,0.77)	0.72 (0.62,0.83)	0.60 (0.52,0.71)	0.57 (0.49,0.67)	0.44 (0.40,0.49)	0.36 (0.32,0.41)	0.21 (0.17,0.27)	0.11 (0.10,0.12)
Model 2[b]	1	0.69 (0.58,0.81)	0.79 (0.69,0.92)	0.83 (0.72,0.96)	0.68 (0.58,0.79)	0.63 (0.54,0.74)	0.46 (0.42,0.51)	0.35 (0.30,0.40)	0.20 (0.16,0.25)	-
Obesity-related cancer[e]										
Case/person-year	2,714/661,933	424/83,155	405/87,769	377/80,180	410/76,795	419/69,624	1,322/215,636	851/123,707	362/49,759	12,136/3,477,419
Model 1[a]	1	1.23 (1.11,1.36)	1.05 (0.94,1.17)	1.02 (0.91,1.13)	1.10 (0.99,1.22)	1.15 (1.04,1.28)	1.02 (0.95,1.09)	1.01 (0.93,1.09)	1.08 (0.96,1.22)	0.94 (0.90,0.98)
Model 2[b]	1	1.27 (1.14,1.41)	1.08 (0.97,1.20)	1.06 (0.95,1.18)	1.13 (1.01,1.25)	1.18 (1.06,1.31)	1.05 (0.98,1.13)	1.03 (0.95,1.13)	1.12 (0.98,1.27)	-
Lung cancer										
Case/person-year	1,640/663,307	115/83,427	165/88,026	171/80,392	143/77,035	141/69,916	392/216,558	224/124,331	55/50,027	489/3,488,189
Model 1[a]	1	0.54 (0.45,0.66)	0.69 (0.58,0.81)	0.73 (0.62,0.86)	0.60 (0.51,0.72)	0.58 (0.49,0.69)	0.42 (0.38,0.48)	0.32 (0.27,0.37)	0.17 (0.13,0.22)	0.06 (0.06,0.07)

Model 2[b]	1	0.67 (0.55,0.81)	0.81 (0.69,0.96)	0.85 (0.72,1.00)	0.68 (0.57,0.80)	0.63 (0.53,0.76)	0.44 (0.39,0.49)	0.30 (0.26,0.35)	0.16 (0.12,0.21)	-
Colorectal cancer										
Case/person-year	483/663,883	78/83,460	69/88,068	96/80,422	71/77,087	71/69,951	228/216,612	137/124,347	47/50,023	1,713/3,487,019
Model 1[a]	1	1.15 (0.90,1.46)	0.95 (0.74,1.23)	1.36 (1.09,1.69)	1.00 (0.78,1.29)	1.08 (0.84,1.38)	1.00 (0.85,1.18)	1.00 (0.82,1.23)	0.95 (0.69,1.30)	0.77 (0.70,0.86)
Model 2[b]	1	1.22 (0.95,1.56)	1.01 (0.78,1.31)	1.48 (1.18,1.86)	1.05 (0.81,1.36)	1.09 (0.84,1.41)	0.96 (0.81,1.14)	0.92 (0.74,1.14)	0.81 (0.57,1.14)	-
Pancreatic cancer										
Case/person-year	161/664,219	13/83,513	28/88,115	17/80,498	16/77,128	28/69,999	69/216,785	38/124,457	18/50,054	480/3,488,325
Model 1[a]	1	0.48 (0.27,0.84)	0.93 (0.62,1.39)	0.60 (0.36,0.99)	0.55 (0.32,0.92)	0.97 (0.64,1.45)	0.67 (0.50,0.89)	0.50 (0.35,0.72)	0.58 (0.35,0.97)	0.52 (0.43,0.63)
Model 2[b]	1	0.56 (0.31,1.01)	1.11 (0.74,1.68)	0.69 (0.41,1.15)	0.63 (0.37,1.07)	1.12 (0.73,1.71)	0.80 (0.59,1.08)	0.58 (0.39,0.86)	0.61 (0.35,1.06)	-
Kidney cancer										
Case/person-year	134/664,169	29/83,497	17/88,115	18/80,486	22/77,121	15/69,998	61/216,759	41/124,445	4/50,057	492/3,488,077
Model 1[a]	1	1.51 (1.01,2.26)	0.82 (0.49,1.36)	0.86 (0.52,1.41)	1.08 (0.68,1.71)	0.78 (0.46,1.34)	0.85 (0.62,1.16)	0.87 (0.60,1.26)	0.25 (0.09,0.68)	0.69 (0.57,0.85)
Model 2[b]	1	1.75 (1.15,2.64)	0.88 (0.53,1.47)	0.97 (0.58,1.60)	1.24 (0.77,1.97)	0.85 (0.49,1.47)	0.95 (0.69,1.32)	0.98 (0.66,1.45)	0.28 (0.10,0.80)	-
Bladder cancer										
Case/person-year	262/664,038	33/83,484	32/88,101	35/80,472	21/77,122	28/69,987	85/216,710	64/124,412	19/50,044	394/3,488,189
Model 1[a]	1	0.73 (0.50,1.05)	0.67 (0.46,0.98)	0.75 (0.53,1.08)	0.46 (0.30,0.73)	0.61 (0.41,0.90)	0.54 (0.42,0.70)	0.61 (0.45,0.81)	0.47 (0.28,0.76)	0.27 (0.23,0.32)
Model 2[b]	1	0.88 (0.61,1.28)	0.78 (0.53,1.13)	0.86 (0.59,1.23)	0.53 (0.33,0.83)	0.70 (0.46,1.04)	0.60 (0.46,0.78)	0.67 (0.49,0.91)	0.50 (0.29,0.84)	-
Postmenopausal breast cancer										
Case/person-year	1,277/618,458	206/66,234	195/71,587	171/65,794	191/64,088	206/58,593	656/184,610	456/111,213	217/49,503	5,820/3,095,383
Model 1[a]	1	1.33 (1.15,1.54)	1.13 (0.97,1.31)	1.02 (0.87,1.20)	1.12 (0.96,1.30)	1.20 (1.03,1.39)	1.04 (0.94,1.15)	1.06 (0.95,1.18)	1.23 (1.05,1.43)	1.03 (0.97,1.10)
Model 2[b]	1	1.38 (1.19,1.61)	1.14 (0.97,1.32)	1.05 (0.90,1.24)	1.14 (0.97,1.33)	1.20 (1.03,1.40)	1.06 (0.96,1.18)	1.08 (0.96,1.22)	1.30 (1.09,1.54)	-
Ovarian cancer										
Case/person-year	217/619,472	20/66,389	29/71,747	23/65,936	17/64,244	17/58,794	78/185,166	51/111,615	26/49,684	857/3,100,249
Model 1[a]	1	0.81 (0.51,1.29)	1.08 (0.73,1.60)	0.87 (0.56,1.34)	0.63 (0.38,1.03)	0.67 (0.41,1.11)	0.87 (0.66,1.13)	0.89 (0.65,1.22)	1.17 (0.76,1.81)	0.87 (0.74,1.02)

Model 2[b]	1	0.79 (0.50,1.27)	1.15 (0.77,1.70)	0.87 (0.56,1.35)	0.64 (0.39,1.06)	0.68 (0.41,1.12)	0.87 (0.66,1.16)	0.90 (0.63,1.27)	1.33 (0.81,2.18)	-
Endometrial cancer										
Case/person-year	262/619,410	48/66,365	37/71,738	34/65,922	52/64,210	43/58,767	138/185,116	70/111,591	38/49,664	1,742/3,099,300
Model 1[a]	1	1.55 (1.14,2.12)	1.09 (0.77,1.54)	1.06 (0.74,1.52)	1.62 (1.20,2.19)	1.37 (0.99,1.90)	1.33 (1.08,1.65)	1.18 (0.90,1.56)	1.69 (1.17,2.42)	1.43 (1.25,1.64)
Model 2[b]	1	1.56 (1.14,2.14)	1.08 (0.76,1.54)	1.06 (0.73,1.52)	1.61 (1.18,2.18)	1.36 (0.97,1.90)	1.38 (1.10,1.73)	1.24 (0.92,1.67)	1.71 (1.12,2.59)	-

[a] Adjusting for age (months), cohort origin (NHS, NHSII, HPFS), race (African American, Asian, Others, White), history of hypertension, history of high cholesterol, family history of cancer, total energy (quintiles), physical activity (quintiles), multivitamin use (yes, no), alcohol intake (None, 1-4 g/d, 5-9 g/d, 10-14 g/d, 15-29 g/d, ≥30 g/d), AHEI (quintiles), and baseline BMI (< 21.0, 21.0-22.9, 23.0-24.9, 25.0-26.9, 27.0-29.9, 30.0-32.9, 33.0-34.9, or ≥ 35.0 kg/m^2). Postmenopausal hormone use was adjusted for women.

[b] Model 1 + cigarettes smoked per day (1-4, 5-14, 15-24, 25-34, 35-44, ≥ 45, unknown), age (years) at starting smoking (< 15, 15-19, 20-29, 30-39, 40-49, 50-59, ≥ 60). Never smokers were excluded in the model.

[c]Total smoking-related cancer includes liver cancer, colorectal cancer, lung cancer, oral cavity and throat cancer, esophagus cancer, larynx cancer, stomach cancer, pancreatic cancer, bladder cancer, kidney cancer, cervix cancer, and acute myeloid leukemia.

[d]Robust smoking-related cancer includes oral cavity and throat cancer, lung cancer, esophagus cancer, and bladder cancer.

[e]Obesity-related cancer includes esophagus cancer, liver cancer, kidney cancer, myeloma, pancreatic cancer, colorectal cancer, gallbladder cancer, postmenopausal breast cancer, ovarian cancer, endometrial cancer, fatal prostate cancer, and thyroid cancer.

Supplementary Table 3. Pooled hazard ratios (95% CIs) of associations between no weight change and weight gain following smoking cessation and risk of cancers.

			Weight gain within 6 years after smoking cessation[a]			
Cancer outcomes	Current smokers	No weight change	>0-5 kg	>5-10 kg	≥10 kg	Never smokers
Total cancer						
Case/person-year	5,927/659,668	569/58,049	2,507/241,476	1,792/161,033	1,366/123,179	20,295/3,470,308
Age-adjusted model	1.00	0.86 (0.79,0.94)	0.88 (0.84,0.92)	0.94 (0.89,0.99)	1.00 (0.94,1.06)	0.69 (0.67,0.71)
Multivariable-adjusted model 1[e]	1.00	0.87 (0.79,0.94)	0.85 (0.81,0.89)	0.90 (0.85,0.95)	0.95 (0.89,1.01)	0.68 (0.66,0.71)
Multivariable-adjusted model 2[f]	1.00	0.87 (0.79,0.95)	0.85 (0.81,0.89)	0.88 (0.83,0.93)	0.93 (0.88,0.99)	
Total smoking-related cancer[b]						
Case/person-year	2,965/662,286	236/58,338	947/242,850	720/161,985	551/123,885	4,247/3,485,136
Age-adjusted model	1.00	0.62 (0.54,0.71)	0.58 (0.54,0.63)	0.67 (0.62,0.73)	0.76 (0.70,0.84)	0.28 (0.27,0.30)
Multivariable-adjusted model 1[e]	1.00	0.64 (0.56,0.73)	0.60 (0.55,0.64)	0.67 (0.62,0.73)	0.75 (0.69,0.83)	0.29 (0.28,0.31)
Multivariable-adjusted model 2[f]	1.00	0.68 (0.60,0.78)	0.61 (0.56,0.66)	0.65 (0.60,0.71)	0.71 (0.65,0.78)	
Robust smoking-related cancer[c]						
Case/person-year	2,039/662,964	138/58,407	537/243,159	429/162,216	304/124,075	1,092/3,487,652
Age-adjusted model	1.00	0.50 (0.42,0.59)	0.44 (0.40,0.49)	0.54 (0.48,0.60)	0.56 (0.50,0.64)	0.10 (0.09,0.11)
Multivariable-adjusted model 1[e]	1.00	0.51 (0.43,0.61)	0.45 (0.41,0.50)	0.55 (0.49,0.61)	0.59 (0.52,0.67)	0.11 (0.10,0.12)
Multivariable-adjusted model 2[f]	1.00	0.58 (0.49,0.69)	0.48 (0.44,0.53)	0.53 (0.47,0.59)	0.53 (0.47,0.61)	
Obesity-related cancer[d]						
Case/person-year	2,714/661,933	331/58,217	1,422/242,306	1,046/161,568	811/123,588	12,136/3,477,419
Age-adjusted model	1.00	1.05 (0.94,1.18)	1.06 (0.99,1.13)	1.15 (1.07,1.23)	1.25 (1.15,1.35)	0.97 (0.93,1.01)
Multivariable-adjusted model 1[e]	1.00	1.05 (0.94,1.18)	1.02 (0.95,1.09)	1.09 (1.01,1.17)	1.15 (1.06,1.24)	0.94 (0.90,0.98)
Multivariable-adjusted model 2[f]	1.00	1.08 (0.96,1.21)	1.04 (0.97,1.12)	1.11 (1.03,1.20)	1.20 (1.10,1.30)	
Lung cancer						
Case/person-year	1,640/663,307	109/58,435	416/243,269	341/162,293	244/124,130	489/3,488,189
Age-adjusted model	1.00	0.48 (0.39,0.58)	0.41 (0.37,0.46)	0.51 (0.45,0.58)	0.54 (0.47,0.62)	0.06 (0.05,0.06)
Multivariable-adjusted model 1[e]	1.00	0.50 (0.41,0.61)	0.42 (0.38,0.48)	0.53 (0.47,0.60)	0.57 (0.50,0.66)	0.06 (0.06,0.07)

Multivariable-adjusted model 2[f]	1.00	0.58 (0.47,0.70)	0.46 (0.41,0.51)	0.51 (0.45,0.58)	0.51 (0.45,0.59)	
Colorectal cancer						
Case/person-year	483/663,883	59/58,451	233/243,371	156/162,383	150/124,148	1,713/3,487,019
Age-adjusted model	1.00	1.04 (0.79,1.37)	0.98 (0.84,1.15)	0.98 (0.82,1.18)	1.45 (1.20,1.75)	0.76 (0.68,0.84)
Multivariable-adjusted model 1[e]	1.00	1.06 (0.81,1.39)	1.00 (0.85,1.17)	0.98 (0.81,1.18)	1.39 (1.15,1.68)	0.77 (0.69,0.86)
Multivariable-adjusted model 2[f]	1.00	1.05 (0.80,1.39)	0.98 (0.82,1.16)	0.94 (0.78,1.14)	1.35 (1.11,1.64)	
Pancreatic cancer						
Case/person-year	161/664,219	20/58,497	65/243,539	50/162,492	41/124,268	480/3,488,325
Age-adjusted model	1.00	0.83 (0.52,1.33)	0.64 (0.48,0.86)	0.77 (0.55,1.06)	0.90 (0.64,1.28)	0.52 (0.43,0.62)
Multivariable-adjusted model 1[e]	1.00	0.84 (0.52,1.34)	0.65 (0.49,0.88)	0.76 (0.55,1.06)	0.86 (0.60,1.22)	0.52 (0.43,0.63)
Multivariable-adjusted model 2[f]	1.00	0.95 (0.59,1.54)	0.72 (0.53,0.99)	0.85 (0.61,1.19)	0.94 (0.66,1.36)	
Kidney cancer						
Case/person-year	134/664,169	9/58,493	60/243,519	49/162,476	30/124,256	492/3,488,077
Age-adjusted model	1.00	0.56 (0.28,1.09)	0.87 (0.64,1.19)	1.05 (0.75,1.46)	0.94 (0.63,1.41)	0.74 (0.60,0.90)
Multivariable-adjusted model 1[e]	1.00	0.55 (0.28,1.09)	0.91 (0.66,1.24)	1.01 (0.72,1.41)	0.77 (0.51,1.15)	0.69 (0.57,0.85)
Multivariable-adjusted model 2[f]	1.00	0.61 (0.31,1.22)	0.97 (0.70,1.35)	1.09 (0.77,1.55)	0.85 (0.56,1.30)	
Bladder cancer						
Case/person-year	262/664,038	22/58,481	91/243,475	67/162,458	44/124,245	394/3,488,189
Age-adjusted model	1.00	0.64 (0.41,1.00)	0.64 (0.50,0.82)	0.72 (0.55,0.95)	0.71 (0.52,0.99)	0.27 (0.23,0.32)
Multivariable-adjusted model 1[e]	1.00	0.63 (0.41,0.98)	0.59 (0.46,0.76)	0.68 (0.52,0.90)	0.69 (0.50,0.96)	0.27 (0.22,0.32)
Multivariable-adjusted model 2[f]	1.00	0.69 (0.44,1.09)	0.64 (0.50,0.83)	0.69 (0.52,0.92)	0.68 (0.48,0.95)	
Postmenopausal breast cancer						
Case/person-year	1,277/618,458	159/53,876	768/227,302	578/154,523	405/119,525	5,820/3,095,383
Age-adjusted model	1.00	1.04 (0.88,1.23)	1.16 (1.06,1.27)	1.27 (1.15,1.41)	1.23 (1.09,1.37)	1.05 (0.99,1.12)
Multivariable-adjusted model 1[e]	1.00	1.06 (0.90,1.25)	1.07 (0.97,1.17)	1.17 (1.06,1.30)	1.15 (1.02,1.29)	1.04 (0.97,1.10)
Multivariable-adjusted model 2[f]	1.00	1.07 (0.90,1.26)	1.10 (1.00,1.21)	1.19 (1.07,1.32)	1.19 (1.06,1.35)	
Ovarian cancer						
Case/person-year	217/619,472	22/54,016	86/227,947	58/155,039	52/119,842	857/3,100,249
Age-adjusted model	1.00	0.95 (0.61,1.48)	0.86 (0.67,1.11)	0.84 (0.62,1.12)	1.10 (0.81,1.50)	0.90 (0.77,1.05)
Multivariable-adjusted model 1[e]	1.00	0.95 (0.61,1.49)	0.83 (0.64,1.08)	0.80 (0.60,1.08)	1.03 (0.76,1.40)	0.87 (0.74,1.01)

Multivariable-adjusted model 2[f]	1.00	0.97 (0.61,1.52)	0.81 (0.61,1.06)	0.80 (0.59,1.09)	1.09 (0.79,1.51)	
Endometrial cancer						
Case/person-year	262/619,410	42/53,999	121/227,894	113/154,989	109/119,788	1,742/3,099,300
Age-adjusted model	1.00	1.62 (1.17,2.25)	1.11 (0.89,1.38)	1.53 (1.22,1.91)	1.99 (1.59,2.50)	1.61 (1.41,1.84)
Multivariable-adjusted model 1[e]	1.00	1.59 (1.15,2.21)	1.13 (0.91,1.41)	1.44 (1.15,1.80)	1.56 (1.24,1.95)	1.43 (1.25,1.64)
Multivariable-adjusted model 2[f]	1.00	1.68 (1.20,2.36)	1.10 (0.87,1.39)	1.44 (1.14,1.81)	1.57 (1.23,1.99)	

[a] Weight change were stopped updating after 6 years of quitting.

[b] Total smoking-related cancer includes liver cancer, colorectal cancer, lung cancer, oral cavity and throat cancer, esophagus cancer, larynx cancer, stomach cancer, pancreatic cancer, bladder cancer, kidney cancer, cervix cancer, and acute myeloid leukemia.

[c] Robust smoking-related cancer includes oral cavity and throat cancer, lung cancer, esophagus cancer, and bladder cancer.

[d] Obesity-related cancer includes esophagus cancer, liver cancer, kidney cancer, myeloma, pancreatic cancer, colorectal cancer, gallbladder cancer, postmenopausal breast cancer, ovarian cancer, endometrial cancer, fatal prostate cancer, and thyroid cancer.

[e] Adjusting for age (months), cohort origin (NHS, NHSII, HPFS), race (White, African American, Asian, Others), history of hypertension, history of high cholesterol, family history of cancer, total energy (quintiles), physical activity (quintiles), multivitamin use (yes, no), alcohol intake (None, 1-4 g/d, 5-9 g/d, 10-14 g/d, 15-29 g/d, ≥30 g/d), AHEI (quintiles), and baseline BMI (< 21.0, 21.0-22.9, 23.0-24.9, 25.0-26.9, 27.0-29.9, 30.0-32.9, 33.0-34.9, or ≥ 35.0 kg/m^2). Postmenopausal hormone use was adjusted for women.

[f] Model 1 + cigarettes smoked per day (1-4, 5-14, 15-24, 25-34, 35-44, ≥ 45, unknown), age (years) at starting smoking (< 15, 15-19, 20-29, 30-39, 40-49, 50-59, ≥ 60). Never smokers were excluded in the model.

Supplementary Table 4. Pooled hazard ratios (95% CIs) of associations between weight loss and weight gain following smoking cessation and risk of cancers.

			Weight gain within 6 years after smoking cessation[a]			
Cancer outcomes	Current smokers	Weight loss	0-5 kg	>5-10 kg	≥10 kg	Never smokers
Total cancer						
Case/person-year	5,927/659,668	1,243/107,110	3,076/299,526	1,792/161,033	1,366/123,179	20,295/3,470,308
Age-adjusted model	1.00	0.97 (0.91,1.03)	0.88 (0.84,0.92)	0.94 (0.89,0.99)	0.99 (0.94,1.05)	0.69 (0.67,0.71)
Multivariable-adjusted model 1[e]	1.00	0.93 (0.87,0.99)	0.85 (0.82,0.89)	0.90 (0.85,0.95)	0.95 (0.89,1.01)	0.68 (0.66,0.71)
Multivariable-adjusted model 2[f]	1.00	0.94 (0.88,1.00)	0.86 (0.82,0.90)	0.88 (0.83,0.93)	0.93 (0.87,0.99)	
Total smoking-related cancer[b]						
Case/person-year	2,965/662,286	560/107,701	1,183/301,188	720/161,985	551/123,885	4,247/3,485,136
Age-adjusted model	1.00	0.73 (0.67,0.80)	0.59 (0.55,0.63)	0.67 (0.61,0.73)	0.76 (0.69,0.83)	0.28 (0.27,0.30)
Multivariable-adjusted model 1[e]	1.00	0.74 (0.67,0.81)	0.61 (0.56,0.65)	0.68 (0.62,0.73)	0.75 (0.68,0.82)	0.29 (0.28,0.31)
Multivariable-adjusted model 2[f]	1.00	0.80 (0.73,0.88)	0.63 (0.58,0.67)	0.65 (0.60,0.71)	0.71 (0.64,0.78)	
Robust smoking-related cancer[c]						
Case/person-year	2,039/662,964	324/107,860	675/301,566	429/162,216	304/124,075	1,092/3,487,652
Age-adjusted model	1.00	0.57 (0.50,0.64)	0.45 (0.41,0.50)	0.54 (0.48,0.60)	0.56 (0.50,0.63)	0.10 (0.09,0.11)
Multivariable-adjusted model 1[e]	1.00	0.59 (0.52,0.67)	0.46 (0.42,0.51)	0.55 (0.49,0.61)	0.58 (0.52,0.66)	0.11 (0.10,0.12)
Multivariable-adjusted model 2[f]	1.00	0.68 (0.60,0.76)	0.50 (0.46,0.55)	0.53 (0.47,0.59)	0.53 (0.47,0.60)	
Obesity-related cancer[d]						
Case/person-year	2,714/661,933	700/107,490	1,753/300,522	1,046/161,568	811/123,588	12,136/3,477,419
Age-adjusted model	1.00	1.16 (1.06,1.26)	1.06 (0.99,1.12)	1.15 (1.07,1.23)	1.25 (1.15,1.35)	0.97 (0.93,1.01)
Multivariable-adjusted model 1[e]	1.00	1.09 (1.00,1.19)	1.02 (0.96,1.09)	1.09 (1.01,1.17)	1.15 (1.06,1.24)	0.94 (0.90,0.99)
Multivariable-adjusted model 2[f]	1.00	1.14 (1.04,1.24)	1.05 (0.98,1.12)	1.11 (1.03,1.20)	1.20 (1.10,1.30)	
Lung cancer						
Case/person-year	1,640/663,307	246/107,934	525/301,703	341/162,293	244/124,130	489/3,488,189
Age-adjusted model	1.00	0.52 (0.45,0.59)	0.42 (0.38,0.47)	0.51 (0.45,0.57)	0.54 (0.47,0.62)	0.06 (0.05,0.06)
Multivariable-adjusted model 1[e]	1.00	0.55 (0.48,0.63)	0.44 (0.40,0.49)	0.53 (0.47,0.60)	0.57 (0.49,0.65)	0.06 (0.06,0.07)
Multivariable-adjusted model 2[f]	1.00	0.64 (0.55,0.74)	0.48 (0.43,0.54)	0.51 (0.45,0.58)	0.51 (0.44,0.59)	

Colorectal cancer						
Case/person-year	483/663,883	139/107,986	292/301,824	156/162,383	150/124,148	1,713/3,487,019
Age-adjusted model	1.00	1.26 (1.04,1.53)	1.00 (0.86,1.16)	0.99 (0.82,1.18)	1.45 (1.20,1.74)	0.76 (0.69,0.84)
Multivariable-adjusted model 1[e]	1.00	1.24 (1.03,1.51)	1.01 (0.87,1.17)	0.98 (0.82,1.18)	1.39 (1.15,1.67)	0.77 (0.69,0.86)
Multivariable-adjusted model 2[f]	1.00	1.27 (1.04,1.55)	1.00 (0.85,1.17)	0.95 (0.79,1.15)	1.34 (1.10,1.63)	
Pancreatic cancer						
Case/person-year	161/664,219	27/108,082	85/302,034	50/162,492	41/124,268	480/3,488,325
Age-adjusted model	1.00	0.55 (0.37,0.83)	0.68 (0.52,0.88)	0.76 (0.55,1.06)	0.91 (0.64,1.29)	0.52 (0.43,0.62)
Multivariable-adjusted model 1[e]	1.00	0.54 (0.36,0.82)	0.69 (0.53,0.91)	0.77 (0.55,1.06)	0.86 (0.61,1.23)	0.52 (0.43,0.63)
Multivariable-adjusted model 2[f]	1.00	0.62 (0.41,0.95)	0.77 (0.58,1.02)	0.84 (0.60,1.17)	0.95 (0.66,1.37)	
Kidney cancer						
Case/person-year	134/664,169	41/108,063	69/302,013	49/162,476	30/124,256	492/3,488,077
Age-adjusted model	1.00	1.28 (0.90,1.83)	0.82 (0.61,1.10)	1.06 (0.76,1.48)	0.95 (0.63,1.41)	0.74 (0.61,0.90)
Multivariable-adjusted model 1[e]	1.00	1.16 (0.81,1.66)	0.84 (0.62,1.13)	1.02 (0.73,1.42)	0.78 (0.52,1.16)	0.70 (0.57,0.85)
Multivariable-adjusted model 2[f]	1.00	1.38 (0.95,2.01)	0.91 (0.67,1.25)	1.12 (0.79,1.58)	0.88 (0.58,1.34)	
Bladder cancer						
Case/person-year	262/664,038	49/108,043	113/301,957	67/162,458	44/124,245	394/3,488,189
Age-adjusted model	1.00	0.70 (0.52,0.96)	0.64 (0.51,0.80)	0.72 (0.55,0.94)	0.71 (0.51,0.99)	0.27 (0.23,0.32)
Multivariable-adjusted model 1[e]	1.00	0.66 (0.49,0.91)	0.60 (0.48,0.75)	0.68 (0.51,0.89)	0.68 (0.49,0.95)	0.26 (0.22,0.31)
Multivariable-adjusted model 2[f]	1.00	0.76 (0.55,1.04)	0.65 (0.51,0.83)	0.69 (0.52,0.91)	0.67 (0.48,0.93)	
Postmenopausal breast cancer						
Case/person-year	1,277/618,458	330/98,700	927/281,178	578/154,523	405/119,525	5,820/3,095,383
Age-adjusted model	1.00	1.12 (0.99,1.27)	1.14 (1.05,1.24)	1.27 (1.15,1.41)	1.22 (1.09,1.37)	1.05 (0.99,1.12)
Multivariable-adjusted model 1[e]	1.00	1.06 (0.94,1.20)	1.06 (0.98,1.16)	1.17 (1.06,1.29)	1.15 (1.02,1.29)	1.03 (0.97,1.10)
Multivariable-adjusted model 2[f]	1.00	1.09 (0.96,1.24)	1.09 (1.00,1.20)	1.19 (1.07,1.32)	1.19 (1.06,1.34)	
Ovarian cancer						
Case/person-year	217/619,472	38/98,980	108/281,963	58/155,039	52/119,842	857/3,100,249
Age-adjusted model	1.00	0.87 (0.62,1.24)	0.87 (0.69,1.10)	0.83 (0.62,1.12)	1.10 (0.81,1.50)	0.90 (0.77,1.05)
Multivariable-adjusted model 1[e]	1.00	0.83 (0.58,1.18)	0.85 (0.67,1.08)	0.80 (0.60,1.08)	1.03 (0.76,1.41)	0.87 (0.74,1.01)
Multivariable-adjusted model 2[f]	1.00	0.84 (0.59,1.21)	0.83 (0.64,1.07)	0.80 (0.59,1.08)	1.11 (0.80,1.53)	

Endometrial cancer						
Case/person-year	262/619,410	60/98,957	163/281,893	113/154,989	109/119,788	1,742/3,099,300
Age-adjusted model	1.00	1.27 (0.95,1.68)	1.21 (0.99,1.47)	1.53 (1.22,1.91)	1.99 (1.59,2.50)	1.61 (1.41,1.84)
Multivariable-adjusted model 1[e]	1.00	1.09 (0.82,1.44)	1.22 (1.00,1.49)	1.44 (1.15,1.81)	1.56 (1.24,1.96)	1.44 (1.25,1.64)
Multivariable-adjusted model 2[f]	1.00	1.11 (0.83,1.50)	1.22 (0.99,1.51)	1.44 (1.14,1.82)	1.58 (1.24,2.01)	

[a] Weight change were stopped updating after 6 years of quitting.

[b] Total smoking-related cancer includes liver cancer, colorectal cancer, lung cancer, oral cavity and throat cancer, esophagus cancer, larynx cancer, stomach cancer, pancreatic cancer, bladder cancer, kidney cancer, cervix cancer, and acute myeloid leukemia.

[c] Robust smoking-related cancer includes oral cavity and throat cancer, lung cancer, esophagus cancer, and bladder cancer.

[d] Obesity-related cancer includes esophagus cancer, liver cancer, kidney cancer, myeloma, pancreatic cancer, colorectal cancer, gallbladder cancer, postmenopausal breast cancer, ovarian cancer, endometrial cancer, fatal prostate cancer, and thyroid cancer.

[e] Adjusting for age (months), cohort origin (NHS, NHSII, HPFS), race (White, African American, Asian, Others), history of hypertension, history of high cholesterol, family history of cancer, total energy (quintiles), physical activity (quintiles), multivitamin use (yes, no), alcohol intake (None, 1-4 g/d, 5-9 g/d, 10-14 g/d, 15-29 g/d, ≥30 g/d), AHEI (quintiles), and baseline BMI (< 21.0, 21.0-22.9, 23.0-24.9, 25.0-26.9, 27.0-29.9, 30.0-32.9, 33.0-34.9, or ≥ 35.0 kg/m^2). Postmenopausal hormone use was adjusted for women.

[f] Model 1 + cigarettes smoked per day (1-4, 5-14, 15-24, 25-34, 35-44, ≥ 45, unknown), age (years) at starting smoking (< 15, 15-19, 20-29, 30-39, 40-49, 50-59, ≥ 60). Never smokers were excluded in the model.

Supplementary figure 1. Flow chart study population exclusion at baseline and during the follow-up. NHS: Nurses' Health Study; HPFS: Health Professionals Follow-up Study.

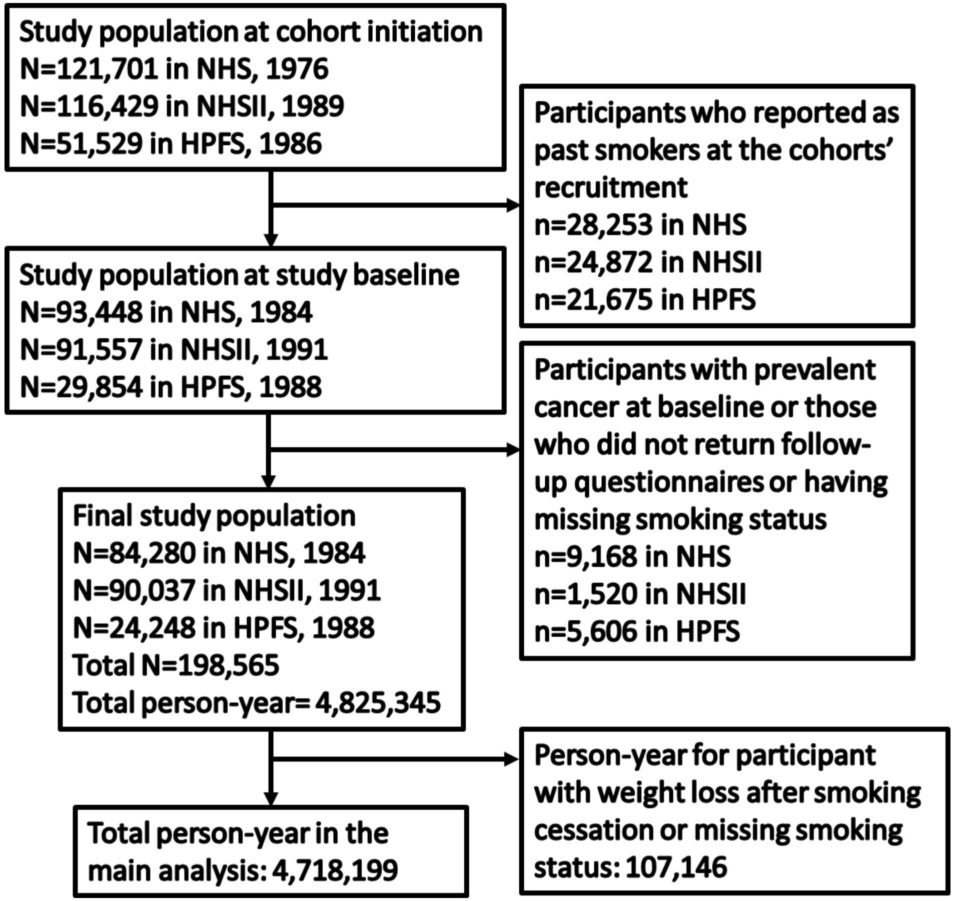

Supplementary figure 2. Association between smoking cessation duration and risk of total cancer (Panel A), total smoking-related cancer (Panel B, robust smoking-related cancer (Panel C), and obesity-related cancer (Panel D), and according to weight gain within 6 years after smoking cessation.

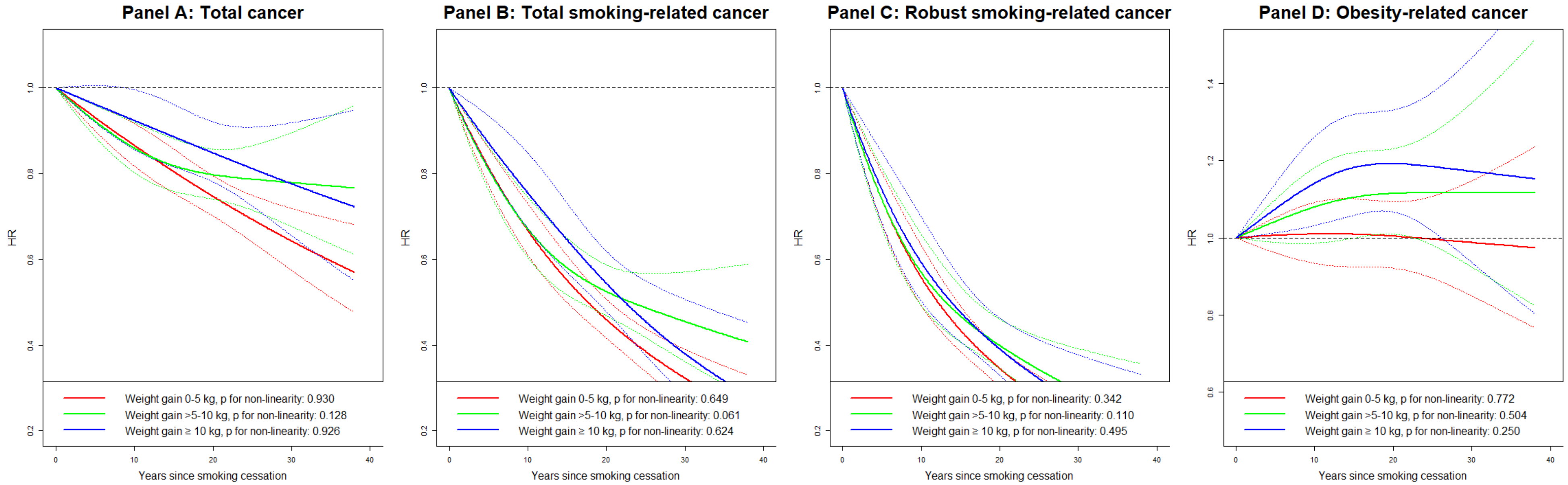

The reference group was the current smokers. Models were age- (months) and calendar-time stratified and adjusted for cohort origin (NHS, NHSII, HPFS), race (African American, Asian, Others, White), history of hypertension, history of high cholesterol, family history of cancer, total energy (quintiles), physical activity (quintiles), multivitamin use (yes, no), alcohol intake (None, 1-4 g/d, 5-9 g/d, 10-14 g/d, 15-29 g/d, ≥30 g/d), AHEI (quintiles), and baseline BMI (< 21.0, 21.0-22.9, 23.0-24.9, 25.0-26.9, 27.0-29.9, 30.0-32.9, 33.0-34.9, or ≥ 35.0 kg/m^2), cigarettes smoked per day (1-4, 5-14, 15-24, 25-34, 35-44, ≥ 45, unknown), age (years) at starting smoking (< 15, 15-19, 20-29, 30-39, 40-49, 50-59, ≥ 60). Total smoking-related cancer includes liver cancer, colorectal cancer, lung cancer, oral cavity and throat cancer, esophagus cancer, larynx cancer, stomach cancer, pancreatic cancer, bladder cancer, kidney cancer, cervix cancer, and acute myeloid leukemia. Robust smoking-related cancer includes oral cavity and throat cancer, lung cancer, esophagus cancer, and bladder cancer. Obesity-related cancer includes esophagus

cancer, liver cancer, kidney cancer, myeloma, pancreatic cancer, colorectal cancer, gallbladder cancer, postmenopausal breast cancer, ovarian cancer, endometrial cancer, fatal prostate cancer, and thyroid cancer. Weight changes were stopped updating after 6 years of quitting. P values for interaction are calculated from 2-side likelihood ratio test and is 0.16 in Panel A, 0.25 in Panel B, 0.70 in Panel C, and 0.25 in Panel D. NHS: Nurses' Health Study; HPFS: Health Professionals Follow-up Study; AHEI: alternative healthy eating index; BMI: body mass index.
